# Determinants of Pregnancy‐Related Misconceptions Among Pregnant Women in Urban Bangladesh: A Cross‐Sectional Study

**DOI:** 10.1002/puh2.70337

**Published:** 2026-08-10

**Authors:** Tasnima Siddiquee, Bishwajit Bhowmik, Sanjida Binte Munir, Hafiza Nasrin, Tanjimul Islam, Purobi Rani Debnath, Fahmida Mahmud, Liya Quamrun Nesa, Tamanna Afroz, Shamima Akhtar, Farsana Rahman Khan, Shamim Khan, Arifur Rahman Khan, Graham A. Hitman, A. K. Azad Khan

**Affiliations:** ^1^ Centre for Global Health Research Diabetic Association of Bangladesh Dhaka Bangladesh; ^2^ Department of Ophthalmology Bangladesh Institute of Research and Rehabilitation in Diabetes Dhaka Bangladesh; ^3^ Blizard Institute Faculty of Medicine and Dentistry Queen Mary University of London London UK

**Keywords:** antenatal care, Bangladesh, health beliefs, maternal knowledge, pregnancy misconceptions, public health, social determinants

## Abstract

**Introduction:**

Pregnancy‐related misconceptions are common in low‐ and middle‐income countries and may adversely influence maternal behaviours. Evidence from urban populations is limited. This study assessed the prevalence and determinants of pregnancy‐related misconceptions among pregnant women in urban Bangladesh.

**Methods:**

A cross‐sectional baseline analysis was conducted among 861 first‐trimester pregnant women (6–14 weeks) from urban and semi‐urban Dhaka, Bangladesh (2014–2015). Only pre‐intervention data from a registered trial were used. A pre‐tested, interviewer‐administered questionnaire assessed nine misconceptions. Correct responses were scored 1 (0–9); ‘do not know’ was scored incorrect. Scores were categorised as poor (0–3), average (4–6) or good (7–9). Multivariable logistic regression identified determinants of good knowledge.

**Results:**

The mean knowledge score was 4.9 ± 2.1. Only 13.8% of participants had good knowledge, whereas 73.3% and 12.9% had average and poor knowledge, respectively. Misconceptions were most common regarding diabetes (65.2%), consumption of oily foods during pregnancy (61.4%) and physical activity (30.5%). Women with more than 10 years of education were significantly more likely to have good knowledge than those with lower educational attainment (adjusted odds ratio [OR] 2.26; 95% confidence interval [CI] 1.41–3.63; *p* = 0.001). No significant associations were observed for age, household income, occupation, religion or area of residence. Sensitivity analyses using continuous knowledge scores produced similar findings.

**Conclusion:**

Pregnancy‐related misconceptions remain widespread among pregnant women in urban Bangladesh. Maternal education is the key determinant of accurate knowledge, underscoring the need for culturally appropriate antenatal education and community‐based interventions.

**Trial Registration:**

ClinicalTrials.gov NCT01924013.

## Graphical abstract

Background

Pregnancy‐related misconceptions may negatively influence maternal behaviours, including diet, physical activity, diabetes management and healthcare‐seeking. Evidence from urban Bangladesh remains limited.

**•** Cross‐sectional baseline analysis of the GIFTS Trial (ClinicalTrials.gov: NCT01924013)

**•** Interviewer‐administered questionnaire assessing nine pregnancy‐related misconceptions

Key Findings

**•** Mean knowledge score: **4.9 ± 2.1** (range 0–9)

^0^ **Good**: 13.8%

^0^ **Average**: 73.3%

^0^ **Poor**: 12.9%

Most Common Misconceptions

**•** Child of a diabetic mother will inevitably develop diabetes (65.2%).

**•** Eating oily foods facilitates smooth delivery (61.4%).

**•** Women with diabetes should not become pregnant (60.7%).

**•** Baby will crumble after birth if mother eats Baim fish (49.0%).

**•** Eating ‘twin foods’ causes twin pregnancy (46.0%).

**•** ‘Do not know’ responses accounted for **22.1%** of all responses.

**•** Uncertainty was highest for dietary and diabetes‐related beliefs.

Women with **>10 years of education** were significantly more likely to have good knowledge: **Adjusted OR = 2.26 (**95% CI 1.41–3.63; *p* = 0.001)

**•** Integrate culturally sensitive counselling into routine antenatal care.

**•** Address misconceptions related to diet, physical activity, diabetes and delivery practices.

**•** Engage family members, particularly mothers‐in‐law and older women.

**•** Strengthen community‐based maternal health education programmes.

Pregnancy‐related misconceptions remain widespread among pregnant women in urban Bangladesh. Maternal education is the strongest determinant of accurate knowledge, highlighting the need for targeted antenatal education and community‐based interventions to improve maternal and neonatal health outcomes.

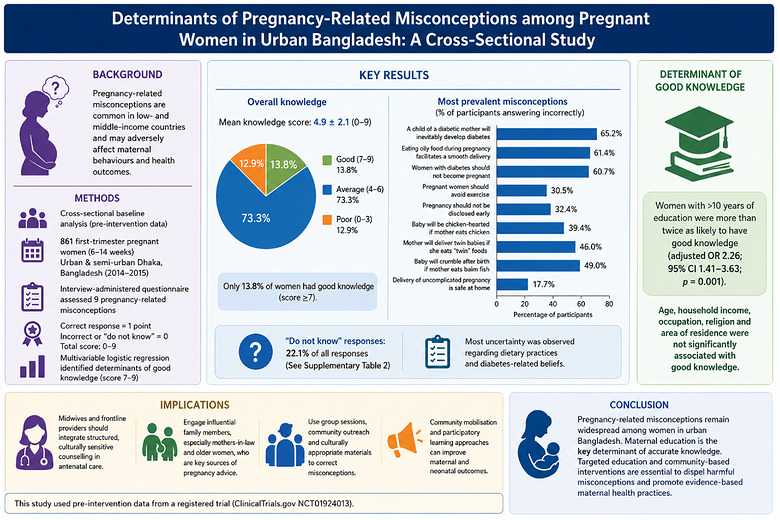

## 1 Introduction

Pregnancy is often accompanied by culturally embedded beliefs, food taboos and misconceptions that have persisted across generations. These beliefs are particularly prominent in low‐ and middle‐income countries (LMICs), where health literacy may be limited and access to formal healthcare services uneven [1, 2]. Such misconceptions may influence maternal behaviours, including dietary practices, physical activity and healthcare‐seeking, potentially leading to adverse maternal and foetal outcomes [3, 4].

Globally, a wide range of culturally driven practices during pregnancy have been documented. In Ethiopia, pregnant women often restrict food intake due to beliefs that excessive eating leads to a large foetus and obstructed labour [5]. In Nigeria, traditional advice discourages consumption of certain foods such as sugar and honey due to perceived risks of prolonged labour [6]. In Southeast Asia, beliefs related to physical behaviours and bodily practices—such as avoiding specific sitting positions or using abdominal bands—are thought to influence foetal positioning and delivery outcomes [7, 8]. These examples highlight the strong influence of cultural norms and intergenerational knowledge on maternal health behaviours.

In South Asia, pregnancy‐related misconceptions are similarly widespread and deeply rooted in social and cultural contexts. In India, for example, foods, such as papaya and pineapple, are often avoided due to perceived risks of miscarriage or foetal harm [9]. These practices are frequently shaped by family members, particularly elders, and may persist despite access to antenatal care and biomedical advice.

In Bangladesh, although maternal health indicators have improved in recent decades, traditional beliefs continue to influence pregnancy‐related practices. Dietary restrictions, often guided by family members, such as mothers‐in‐law, are commonly based on fears of miscarriage, abnormal foetal development or delivery complications [10]. For instance, excessive food intake during pregnancy is sometimes believed to result in a large foetus and difficult or costly caesarean delivery [11]. Evidence suggests that many women reduce food intake during pregnancy due to such beliefs, despite increased nutritional requirements [10, 12]. These misconceptions may undermine the effectiveness of antenatal counselling and public health interventions.

Rapid urbanisation increased access to health services, and expanding educational opportunities have transformed the social and healthcare landscape of Bangladesh. Consequently, women living in urban and semi‐urban settings are often assumed to have better access to health information and greater exposure to biomedical messages than their rural counterparts. However, cultural beliefs and family influences may persist despite improved healthcare access, potentially affecting pregnancy‐related behaviours and decision‐making. Understanding whether misconceptions remain prevalent in urban populations is therefore important for designing effective maternal health interventions in increasingly urbanised settings.

Importantly, misconceptions are not limited to diet but also extend to physical activity and pregnancy‐related conditions such as gestational diabetes mellitus. Such beliefs may discourage healthy behaviours, delay care‐seeking and contribute to preventable complications. Previous studies in Bangladesh have primarily focused on food taboos and dietary restrictions in rural or indigenous populations [10, 11]. In contrast, less attention has been paid to misconceptions related to physical activity, diabetes and delivery practices, particularly among urban pregnant women.

The present study focused on nine commonly reported pregnancy‐related misconceptions encompassing dietary practices, physical activity, diabetes and delivery‐related beliefs. These domains were selected because of their potential influence on maternal nutrition, health‐seeking behaviour, pregnancy management and birth outcomes.

Despite the potential public health implications of such beliefs, quantitative evidence regarding the prevalence and determinants of pregnancy‐related misconceptions among urban and semi‐urban pregnant women in Bangladesh remains limited. Furthermore, little is known about the socio‐demographic factors associated with accurate knowledge in these settings. Addressing this evidence gap is important for informing culturally appropriate antenatal education strategies and community‐based interventions. Therefore, this study aimed to assess the prevalence of pregnancy‐related misconceptions and identify their socio‐demographic determinants among pregnant women residing in urban and semi‐urban areas of Dhaka, Bangladesh.

## 2 Methods

### 2.1 Study Design

This cross‐sectional baseline analysis used data collected as part of the baseline visit of the EU‐funded GIFTS clinical trial (Genomic and Lifestyle Predictors of Foetal Outcome Relevant to Diabetes and Obesity and their Relevance to Prevention Strategies in South Asian Peoples). Data were collected at the Maternal and Child Health Training Institute (MCHTI), a tertiary‐level government antenatal care facility in Dhaka, Bangladesh, from August 2014 to October 2015—before any intervention or randomisation. Only pre‐intervention (baseline) data were analysed.

### 2.2 Participants and Recruitment

Pregnant women residing in five administrative areas of Dhaka city (Azimpur, Lalbag, Hazaribag, Kamrangirchar and Keraniganj) were identified through monthly household visits conducted by government Family Welfare Visitors (FWVs). Pregnancies were first identified via history of missed menses and subsequently confirmed by urine pregnancy testing. Eligible women were invited to attend MCHTI for antenatal registration and baseline assessment. Recruitment procedures have been described previously [13].

### 2.3 Eligibility Criteria and Sample Size

Women were eligible if they were aged 18–28 years, at 6–14 weeks’ gestation, had a singleton pregnancy conceived without fertility treatment and provided written informed consent. The age restriction of 18–28 years was determined by the eligibility criteria of the parent GIFTS trial and was not specific to the objectives of the present analysis. Gestational age was calculated from the last menstrual period and verified by ultrasonography; when discrepancies existed, the ultrasound estimate was used. Women who declined participation were excluded. Convenience sampling was used, reflecting the pragmatic recruitment strategy of the parent trial. Between August 2014 and October 2015, 915 women were approached, and 861 consented and completed the baseline assessments. The sample size reflected the number of women enrolled during the recruitment period rather than an a priori calculation.

### 2.4 Data Collection and Measures

A structured questionnaire (Table S1) was administered face‐to‐face by a trained team comprising one physician and three dietitians. Interviews were conducted in Bangla in a private setting to ensure confidentiality. Data captured included socio‐demographic characteristics (age, education, occupation, monthly household expenditure, area of residence and religion) and pregnancy‐related misconceptions. Monthly household expenditure was categorised as low (<5000 Bangladeshi Taka [BDT]), medium (5000–10,000 BDT) or high (>10,000 BDT), reflecting the 2014–2015 economic context. Education was categorised as 0–5 years (primary level or below), 6–10 years (secondary level, corresponding to completion of the Secondary School Certificate [SSC]), and >10 years (education beyond secondary school). Area of residence was coded as semi‐urban or urban according to administrative definitions.

The questionnaire was developed using pregnancy‐related beliefs commonly reported in Bangladesh and other South Asian settings and was reviewed by investigators with expertise in maternal health, nutrition and epidemiology. Prior to data collection, the questionnaire was pretested among pregnant women from a similar population who were not included in the final study to assess clarity, comprehension and cultural appropriateness. Minor modifications to wording were made following pre‐testing.

All interviewers received intensive training; training covered standardised questionnaire administration, interviewing techniques, maintenance of confidentiality, minimisation of interviewer bias and appropriate handling of culturally sensitive questions related to pregnancy beliefs. Completed questionnaires were reviewed daily for completeness and accuracy. Data were double‐entered and cross‐checked.

### 2.5 Assessment of Pregnancy‐Related Misconceptions

The nine items were selected because they represented commonly reported beliefs related to maternal nutrition, physical activity, diabetes and delivery practices, all of which have potential implications for maternal and neonatal health.

Each correct response was assigned a score of 1; incorrect or ‘do not know’ responses were scored 0. Negatively framed statements were reverse‐coded so that higher scores indicated fewer misconceptions. The total score ranged from 0 to 9.

‘Do not know’ responses were treated as incorrect responses because the primary objective was to assess accurate knowledge regarding pregnancy‐related beliefs. The frequency of ‘do not know’ responses was analysed separately and is presented in Table S2.

As the questionnaire was designed as a culturally specific knowledge assessment tool rather than a psychometric scale (e.g., Cronbach's alpha), formal assessments of construct validity and internal consistency reliability were not performed. This limitation is acknowledged in Section 4.

For descriptive purposes, scores were categorised on the basis of conceptual thresholds and score distribution into poor (0–3), average (4–6) and good (7–9) knowledge. These thresholds were selected a priori to facilitate interpretation, with scores of 0–3 indicating correct responses to fewer than one‐third of items, scores of 4–6 indicating moderate knowledge and scores of 7–9 indicating good knowledge. For regression analyses, scores were dichotomised into good (7–9) versus not good (0–6).

### 2.6 Statistical Analysis

Analyses were performed using PASW Statistics 21 (IBM Corp., Chicago, IL, USA). Continuous variables are presented as mean ± standard deviation (SD) and categorical variables as frequencies and percentages. Differences in mean knowledge scores across participant characteristics were assessed using independent sample *t*‐tests or one‐way analysis of variance (ANOVA), with homogeneity of variances evaluated by Levene's test (reported in Table 3). Associations between categorical variables were examined using *χ* ^2^ tests. There were no missing data for the variables included in the analyses; therefore, complete‐case analyses were performed.

To identify factors associated with good knowledge, a multivariable logistic regression model was fitted with good knowledge (score ≥7) as the dependent variable. All socio‐demographic variables listed in Table 1—age category, education, occupation, monthly household expenditure, area of residence and religion—were entered simultaneously into the model. Adjusted odds ratios (ORs) with 95% confidence intervals (CIs) are reported. Model fit was assessed using the Hosmer–Lemeshow goodness‐of‐fit test and pseudo *R* ^2^ (Nagelkerke).

**TABLE 1:** Characteristics of the study participants (*n* = 861).

Characteristic	Descriptive measure
Age (years)	22.5 ± 4.2
Age categories, % (*n*)	
<25 years	67.7 (583)
≥25 years	32.3 (278)
Socioeconomic status, % (*n*)	
Low to middle income	46.1 (397)
High income	53.9 (464)
Education, % (*n*)	
0–10 years	82.8 (713)
>10 years	17.2 (148)
Occupation, % (*n*)	
Housewife	87.7 (755)
Other	12.3 (106)
Area of residence, % (*n*)	
Semi‐urban	63.6 (548)
Urban	36.4 (313)
Religion, % (*n*)	
Muslim	96.6 (832)
Others	3.4 (29)
Knowledge score (0–9)	4.9 ± 2.1

*Note:* Data are presented as mean ± standard deviation (SD) or % (*n*), as appropriate. Low‐ to middle‐income households were defined as monthly household expenditure ≤10,000 Bangladeshi Taka (BDT) and high‐income households as >10,000 BDT. Education categories shown in the table were collapsed into ≤10and >10 years of schooling for descriptive and regression analyses; >10 years indicates education beyond secondary level. Area of residence was classified according to administrative definitions. Knowledge scores ranged from 0 to 9, with higher scores indicating fewer pregnancy‐related misconceptions.

To evaluate the robustness of findings and minimise potential information loss associated with dichotomisation of the knowledge score, a sensitivity analysis was performed using multivariable linear regression with the continuous knowledge score (0–9) as the dependent variable. The same covariates included in the logistic regression model were entered simultaneously into the linear regression model. Regression coefficients (*β*) with 95% CIs are reported, and findings are presented in Table S3. Two‐sided *p* values <0.05 were considered statistically significant.

## 3 Results

### 3.1 Participant Characteristics

A total of 861 pregnant women in their first trimester were included in the analysis. The mean age of participants was 22.5 ± 4.2 years, with 67.7% aged <25 years. More than half of participants belonged to high‐income households (53.9%), whereas 46.1% were from low‐ to middle‐income households.

Most women had ≤10 years of formal education (82.8%), and the majority were housewives (87.7%). Nearly two‐thirds of participants resided in semi‐urban areas (63.6%), and the vast majority were Muslim (96.6%). The mean knowledge score was 4.9 ± 2.1. Detailed participant characteristics are presented in Table 1.

### 3.2 Prevalence of Pregnancy‐Related Misconceptions

Pregnancy‐related misconceptions were common across all assessed domains, including food‐related beliefs, physical activity, diabetes and delivery practices.

Regarding food‐related beliefs, approximately half of participants believed that consuming Baim fish (Indian mottled eel) could harm the baby (49.0%), and 46.0% believed that eating twin foods (e.g., twin bananas or twin egg yolks) could result in twin pregnancies. Nearly two‐fifths believed that eating chicken could negatively affect the baby (39.4%), whereas 61.4% believed that consuming oily food during pregnancy facilitates a smooth delivery.

Misconceptions extended beyond dietary practices. Almost one‐third of women believed that pregnancy should not be disclosed during the early months (32.4%), and 30.5% believed that pregnant women should avoid physical exercise. Diabetes‐related misconceptions were also prominent, with 60.7% believing that women with diabetes should not become pregnant and 65.2% believing that a child born to a diabetic mother will inevitably develop diabetes.

**FIGURE 1:**
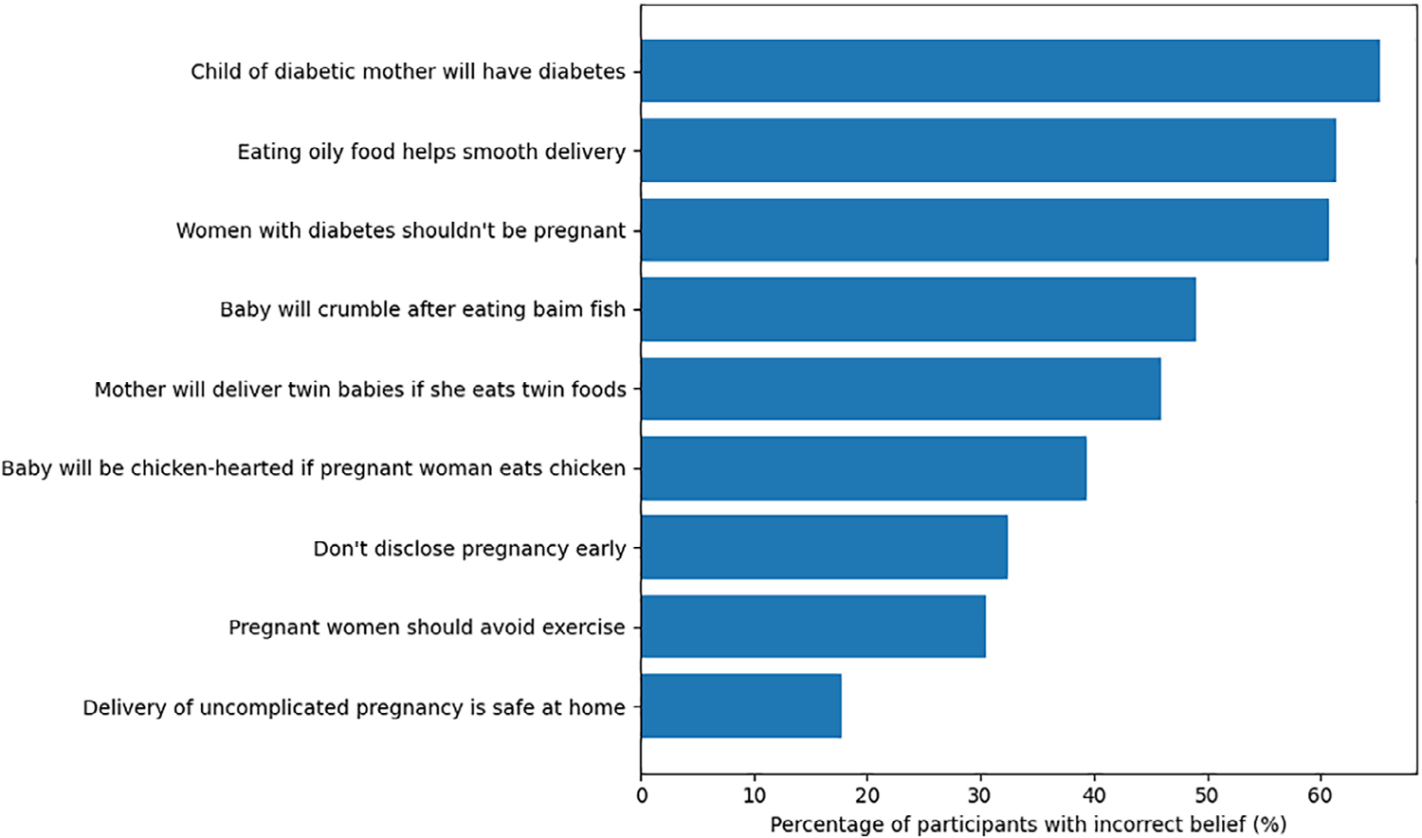
Prevalence of pregnancy‐related misconceptions. Percentage of women who answered each statement incorrectly (*n* = 861). Misconceptions are ordered from most to least prevalent.

In contrast, most participants (82.3%) correctly identified that delivery of an uncomplicated pregnancy is unsafe at home. The distribution of responses is presented in Table 2, and the prevalence of misconceptions is illustrated in Figure 1.

**TABLE 2:** Pregnancy‐related misconceptions (*n* = 861).

Misconception statement	Correct response	% Correct (*n*)	% Incorrect (*n*)
Baby will crumble after birth if mother eats Baim fish	No	51.0 (439)	49.0 (422)
Mother will deliver twin babies if she eats ‘twin’ foods (e.g., twin fruit or twin egg yolks)	No	54.0 (465)	46.0 (396)
Baby will be chicken‐hearted if pregnant woman eats chicken	No	60.6 (522)	39.4 (339)
Pregnancy should not be disclosed during the first few months	No	67.6 (582)	32.4 (279)
Eating oily food during pregnancy facilitates a smooth delivery	No	38.6 (332)	61.4 (529)
Pregnant women should avoid exercise	No	69.5 (598)	30.5 (263)
Women with diabetes should not become pregnant	No	39.3 (338)	60.7 (523)
A child of a diabetic mother will inevitably develop diabetes	No	34.8 (300)	65.2 (561)
Delivery of an uncomplicated pregnancy is safe at home	No	82.3 (709)	17.7 (152)

*Note:* Data are presented as % (*n*). Correct responses indicate the absence of the misconception. Percentages shown for correct and incorrect responses were calculated after combining incorrect and ‘do not know’ responses into a single category. The frequency of ‘do not know’ responses for each item is presented separately in Table S2.

### 3.3 Frequency of ‘Do Not Know’ Responses

Across the nine misconception items, 1712 of 7749 possible responses (22.1%) were recorded as ‘do not know’, indicating substantial uncertainty regarding pregnancy‐related beliefs among participants. The frequency of uncertainty varied considerably across items, with higher levels observed for questions related to dietary practices and diabetes, whereas uncertainty was relatively uncommon for questions concerning pregnancy disclosure and delivery practices. Detailed item‐specific frequencies are presented in Table S2.

### 3.4 Knowledge Score Distribution

**FIGURE 2:**
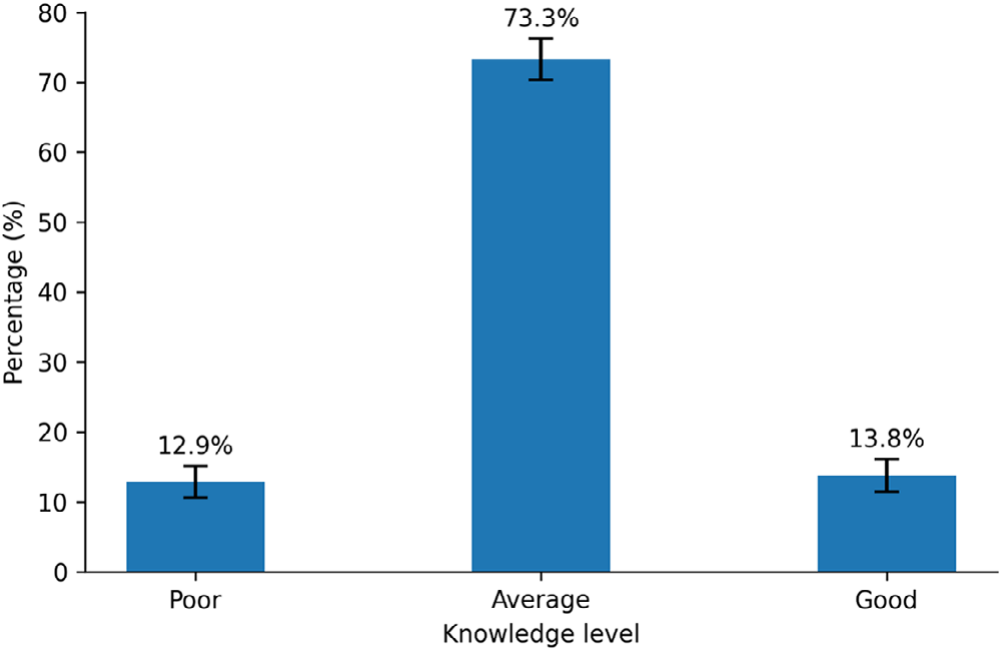
Distribution of pregnancy‐related knowledge levels among pregnant women. Bars represent the percentage of participants classified as having poor, average or good knowledge. Error bars indicate 95% confidence intervals (CI). Percentage values displayed above each bar correspond to the observed prevalence in each knowledge category.

Knowledge scores ranged from 0 to 9, with a mean score of 4.9 ± 2.1. On the basis of predefined cut‐offs, 13.8% of women demonstrated good knowledge (score 7–9), 73.3% had average knowledge (score 4–6), and 12.9% had poor knowledge (score 0–3). The distribution of knowledge levels is shown in Figure 2.

### 3.5 Knowledge Scores and Proportion of Women With Good Knowledge by Participant Characteristics

Table 3 presents mean knowledge scores and the proportion of women with good knowledge according to participant characteristics. Educational attainment was the only characteristic significantly associated with knowledge. Women with more than 10 years of education had higher mean knowledge scores than those with ≤10 years of education (5.92 vs. 4.78; *p* < 0.001) and were more likely to have good knowledge (22.3% vs. 12.1%; *p* = 0.001). No significant differences were observed according to age, socioeconomic status, occupation, area of residence or religion.

**TABLE 3:** Knowledge scores and proportion of women with good knowledge by participant characteristics.

Participant characteristic	Mean score (95 % CI)	*p* value for mean^a^	% With good knowledge (*n*)	*p* value for proportion^b^
Age				
<25 years	5.02 (4.85–5.20)		15.1 (88)	
≥25 years	4.88 (4.64–5.12)	0.35	11.2 (31)	0.12
Socioeconomic status				
Low to middle income	4.96 (4.75–5.18)		14.4 (57)	
High income	4.99 (4.80–5.18)	0.85	13.4 (62)	0.67
Education				
0–10 years	4.78 (4.63–4.94)		12.1 (86)	
>10 years	5.92 (5.61–6.23)	<0.001	22.3 (33)	0.001
Occupation				
Housewife	4.95 (4.80–5.10)		13.8 (104)	
Others	5.15 (4.74–5.56)	0.37	14.2 (15)	0.92
Area of residence				
Semi‐urban	4.93 (4.74–5.11)		13.5 (74)	
Urban	5.07 (4.83–5.30)	0.35	14.4 (45)	0.72
Religion				
Muslim	4.98 (4.84–5.13)		14.2 (118)	
Others	4.76 (4.00–5.52)	0.58	3.4 (1)	0.10

Abbreviation: CI, confidence interval.

^a^ *p* values for mean differences calculated by *t*‐test or one‐way ANOVA, as appropriate.

^b^ *p* values for differences in proportion of women with good knowledge calculated by *χ* ^2^ test.

### 3.6 Factors Associated With Good Knowledge

Multivariable logistic regression analysis identified education as the only independent factor associated with good knowledge of pregnancy‐related misconceptions (Table 4). Women with >10 years of education were more than twice as likely to have good knowledge compared with those with ≤10 years of education (adjusted OR 2.26; 95% CI 1.41–3.63; *p* = 0.001).

**TABLE 4:** Multivariable logistic regression for factors associated with good knowledge (score ≥7).

Variable	Adjusted OR (95 % CI)	*p* value
Age (≥25 vs. <25 years)	0.72 (0.46, 1.11)	0.14
Socioeconomic status (high vs. low‐middle)	0.81 (0.54, 1.21)	0.31
Education (>10 vs. ≤10 years)	2.26 (1.41, 3.63)	0.001
Occupation (housewife vs. other)	1.06 (0.59, 1.94)	0.83
Area of residence (urban vs. semi‐urban)	1.04 (0.69, 1.58)	0.83
Religion (Muslim vs. other)	4.89 (0.65, 36.9)	0.12

*Note:* All variables in Table 1 were entered simultaneously into the model. Model fit: Hosmer–Lemeshow *p* = 0.65; Nagelkerke *R* ^2^ = 0.06.

Abbreviations: CI, confidence interval; OR, odds ratio.

Age, socioeconomic status, occupation, area of residence and religion were not significantly associated with good knowledge after adjustment.

### 3.7 Sensitivity Analysis Using Continuous Knowledge Score

Sensitivity analyses using multivariable linear regression with the continuous knowledge score yielded findings consistent with the primary logistic regression model (Table S3). Women with more than 10 years of education had significantly higher knowledge scores than those with ≤10 years of education (*β* = 1.17, 95% CI 0.79–1.56; *p* < 0.001). No other variables were significantly associated with knowledge score.

## 4 Discussion

This study demonstrates that pregnancy‐related misconceptions persist among women living in urban and semi‐urban Dhaka, despite comparatively good access to antenatal care. Although most participants achieved a moderate score, only a small fraction (13.8%) attained good knowledge (score: 7–9), and the mean score was 4.9 ± 2.1, indicating a generally modest level of understanding. Our multivariable analysis confirmed that maternal education is the sole independent predictor of good knowledge; age, household income, occupation, religion and area of residence showed no significant associations. These findings suggest that entrenched cultural beliefs transcend conventional socio‐demographic boundaries and that education beyond the secondary level plays a pivotal role in countering myths.

Food‐related misconceptions were highly prevalent. Nearly half of women believed that consuming certain foods—such as Baim fish (49.0%) or twin‐yolk eggs (46.0%)—could harm the foetus or induce twin pregnancies, and more than three‐fifths (61.4%) thought oily foods ease childbirth. These findings suggest that food‐related misconceptions remain common across both urban and rural settings in Bangladesh, although the specific foods and perceived consequences may differ between populations. Previous studies from rural Bangladesh have reported avoidance of nutrient‐rich foods because of concerns regarding miscarriage, congenital abnormalities or undesirable characteristics in the newborn [10, 11]. Similar food‐related beliefs have also been documented in Ethiopia, Nigeria and Indonesia [5, 6, 14]. Such restrictions may jeopardise maternal nutrition during a period of increased metabolic demand.

Misconceptions about disclosure and physical activity were also prominent—about one‐third believed pregnancy should remain secret in early months (32.4%) and that women should avoid exercise (30.5%). Comparable concerns regarding physical activity during pregnancy have been reported in Bangladesh, Ethiopia and high‐income settings, where fears of miscarriage or foetal harm may discourage exercise during pregnancy [5, 11, 15, 16]. Sedentary behaviour during pregnancy is linked to adverse outcomes such as gestational diabetes, hypertensive disorders and postpartum depression; correcting these misconceptions should, therefore, be an integral component of antenatal counselling.

Diabetes‐related misconceptions were among the most striking findings. Over 60% believed that women with diabetes should not conceive and that children born to diabetic mothers will inevitably develop diabetes. Similar perceptions have been described in Malaysia [8], where diabetes is viewed as hereditary and inevitably harmful. Such beliefs could foster stigma, delay preconception counselling and discourage women with diabetes from seeking appropriate antenatal care.

An additional finding was the high frequency of ‘do not know’ responses, which accounted for more than one‐fifth of all responses. This suggests that uncertainty regarding pregnancy‐related beliefs is common and that knowledge gaps coexist with misconceptions. Uncertainty was particularly evident for dietary and diabetes‐related items, highlighting areas where women may lack confidence in their understanding of pregnancy‐related health information. Women who are uncertain may be especially responsive to targeted educational interventions because they may not yet hold strongly established misconceptions.

Conversely, most participants (82.3%) correctly recognised that home delivery is unsafe for uncomplicated pregnancies. Although encouraging, awareness of delivery‐related risks does not necessarily translate into timely preparation for facility‐based delivery. Women may recognise the benefits of skilled birth attendance yet still delay arranging transport, finances or family support for facility delivery. Previous studies from Bangladesh and Sub‐Saharan Africa have shown that home delivery remains common because of social norms, financial barriers and limited access to services [17, 18].

Education emerged as the most influential determinant of knowledge. Women with more than 10 years of schooling scored nearly one point higher and were twice as likely to demonstrate good knowledge compared with those with less education. This aligns with findings from Ethiopia and Nigeria [5, 6], where lower educational attainment correlated with adherence to harmful myths. That income and urban residence did not predict knowledge suggests that structural access alone cannot counter entrenched beliefs without targeted education.

These results have important implications for midwifery and public health practice. Midwives and other frontline healthcare providers are often the first trusted contact for pregnant women, giving them a unique opportunity to identify and correct misconceptions early. Routine antenatal counselling should incorporate structured, culturally sensitive discussions about diet, physical activity, diabetes and delivery practices rather than assuming baseline understanding. Engaging influential family members—particularly mothers‐in‐law and older women—is crucial because they are primary sources of pregnancy advice. Midwife‐led group sessions, community outreach and culturally appropriate educational materials can help dismantle misinformation. Community mobilisation and participatory learning approaches, which empower women to identify and address their own knowledge gaps, have proven effective for improving maternal and neonatal outcomes [19]. Policies should, therefore, integrate such interventions into urban and semi‐urban antenatal programmes.

### 4.1 Strengths and Limitations

Strengths of this study include its large sample size and focus on first‐trimester women, enabling early identification of misconceptions. Integration within a public antenatal facility and use of a pretested, culturally adapted questionnaire administered by trained health professionals enhance internal validity. Recruiting participants through household visits also improved community engagement.

However, several limitations should be acknowledged. The cross‐sectional design precludes causal inference and captures beliefs at a single time point. Participants were recruited from women willing to attend antenatal registration at a government facility and may, therefore, represent women who were more engaged with healthcare services than the general pregnant population, introducing the possibility of selection bias. Convenience sampling and inclusion of only urban and semi‐urban centres limit generalisability to rural populations. Furthermore, the study was restricted to women aged 18–28 years because of the eligibility criteria of the parent GIFTS trial, which may limit the applicability of findings to older pregnant women.

The data were collected between 2014 and 2015, and maternal health services, educational attainment and access to digital health information have expanded substantially in Bangladesh since then. Consequently, the prevalence of some misconceptions may have changed over time. Nevertheless, cultural beliefs are often transmitted through families and communities and may persist despite improvements in access to information, supporting the continuing relevance of these findings.

Data were self‐reported, introducing potential recall and social desirability bias. Although the questionnaire was developed from commonly reported beliefs and was pretested before use, formal psychometric validation, including assessment of construct validity and internal consistency reliability, was not undertaken. Finally, the predominance of Muslim participants reflects the study setting and limits assessment of religious differences. Despite these limitations, our findings provide valuable insight into the persistence of pregnancy‐related misconceptions in urban Bangladesh and underscore the centrality of education in addressing them.

## 5 Conclusion

Pregnancy‐related misconceptions remain widespread among women in urban and semi‐urban Dhaka, spanning diet, physical activity, diabetes and delivery practices. In addition to misconceptions, substantial knowledge gaps were identified, particularly regarding dietary and diabetes‐related issues. Although awareness of some issues is encouraging, overall knowledge is moderate, and education beyond the secondary level is the only factor strongly associated with good knowledge. These findings emphasise the need for education‐sensitive, culturally tailored interventions—delivered by midwives and integrated into community outreach—to dispel harmful misconceptions, address knowledge gaps, promote evidence‐based practices and improve maternal and neonatal health outcomes in Bangladesh and similar contexts.

## Author Contributions

**Tasnima Siddiquee**: conceptualisation, data curation, investigation, writing – original draft preparation. **Bishwajit Bhowmik**: project administration, conceptualisation, data curation, formal analysis, investigation, methodology, validation, visualisation, writing – original draft preparation. **Sanjida Binte Munir**: methodology, validation, resources, supervision, writing – review. **Hafiza Nasrin**: project administration, methodology, validation, resources, supervision, writing – review and editing. **Tanjimul Islam**: methodology, validation, resources, supervision. **Purobi Rani Debnath**: writing – review. **Fahmida Mahmud**: data curation, investigation. **Liya Quamrun Nesa**: data curation, investigation, methodology. **Tamanna Afroz**: data curation, investigation, methodology. **Shamima Akhtar**: project administration, data curation. **Farsana Rahman Khan**: data curation, investigation. **Shamim Khan**: data curation, supervision. **Arifur Rahman Khan**: project administration, investigation. **Graham A. Hitman**: conceptualisation, writing – review. **A. K. Azad Khan**: conceptualisation, writing – review.

## Funding

This study was financially supported by the European Union (FP7 EU Grant No. 83599025).

## Ethics Statement

The study protocol conforms to the ethical guidelines of the 1975 Declaration of Helsinki, as reflected in a priori approval by the Norwegian Medical Ethical Committee (East) (2013/845/REK South‐East D) and the Ethical Review Committee of BADAS (BADAS‐ERC/EC/13/00175).

## Consent

Informed consent was obtained from each participant.

## Conflicts of Interest

The authors declare no conflicts of interest.

## Supporting information




**Supplementary Table 1**: Questionnaire used for data collection.
**Supplementary Table 2**: Frequency of “do not know” responses to pregnancy‐related misconception statements (*n* = 861).
**Supplementary Table 3**: Multivariable linear regression analysis of factors associated with total knowledge score (09).

## Data Availability

The datasets used and/or analysed during the current study are available from the corresponding author on reasonable request.
