# Supplementary material for: Determinants of Pregnancy‐Related Misconceptions Among Pregnant Women in Urban Bangladesh: A Cross‐Sectional Study
Source: Public Health Chall. 2026 Aug 10;5(3):e70337. doi: 10.1002/puh2.70337 (PMC13455545; doi:10.1002/puh2.70337)
Supplement: Supplementary file 1 [file PUH2-5-e70337-s001.docx]

| **Supplementary Table** | **Title** | **Page** |
| --- | --- | --- |
| Supplementary Table 1 | Questionnaire used for data collection | 2 |
| Supplementary Table 2 | Frequency of "do not know" responses to pregnancy-related misconception statements (n = 861) | 3 |
| Supplementary Table 3 | Multivariable linear regression analysis of factors associated with total knowledge score (0–9) | 4 |

**Supplementary Table 1 | Questionnaire used for data collection**

1. Baby will crumble after birth if mother eats Baim fish
a) Yes b) no c) do not know

2. Mother will deliver twin babies if she eat twin food (such as twin fruit or twin egg yolk) during pregnancy
a) Yes b) no c) do not know

3. Baby will be chicken hearted if pregnant woman eat chicken
a) Yes b) no c) do not know

4. First few months of pregnancy should not be disclosed
a) Yes b) no c) do not know

5. Eating oily food during pregnancy helpful for smooth delivery
a) Yes b) no c) do not know

6. Pregnant woman should avoid exercise
a) Yes b) no c) do not know

7. Women with diabetes should not be pregnant
a) Yes b) no c) do not know

8. Child of a diabetic mother will always have diabetes
a) Yes b) no c) do not know

9. Delivery of uncomplicated pregnancy is safe at home
a) Yes b) no c) do not know

| **Supplementary Table 2 \| Frequency of "do not know" responses to pregnancy-related misconception statements (n = 861)** | | |
| --- | --- | --- |
| **Item** | **Misconception Statement** | **"I Don't Know" n (%)** |
| KP1 | Baby will crumble after birth if mother eats baim fish | 131 (15.2%) |
| KP2 | Mother will deliver twin babies if she eats "twin" foods | 69 (7.9%) |
| KP3 | Baby will be chicken-hearted if pregnant woman eats chicken | 298 (34.6%) |
| KP4 | Pregnancy should not be disclosed during the first few months | 52 (6.0%) |
| KP5 | Eating oily food during pregnancy facilitates a smooth delivery | 439 (51.0%) |
| KP6 | Pregnant women should avoid exercise | 111 (12.9%) |
| KP7 | Women with diabetes should not become pregnant | 271 (31.5%) |
| KP8 | A child of a diabetic mother will inevitably develop diabetes | 303 (35.2%) |
| KP9 | Delivery of an uncomplicated pregnancy is safe at home | 38 (3.3%) |
| **Overall** | **All items combined (n=861 × 9 = 7,749 responses)** | **1712 (22.1%)** |

| **Supplementary Table 3 \| Multivariable linear regression analysis of factors associated with total knowledge score (0–9)** | | |
| --- | --- | --- |
| **Variable** | **β coefficient (95% CI)** | **p-value** |
| Age (≥25 vs <25 years) | -0.16 (-0.46, 0.15) | 0.311 |
| Socioeconomic status (high vs low-middle) | -0.13 (-0.42, 0.15) | 0.361 |
| Education (>10 vs ≤10 years) | 1.17 (0.79, 1.56) | <0.001 |
| Occupation (housewife vs other) | -0.06 (-0.49, 0.37) | 0.767 |
| Area of residence (urban vs semi-urban) | 0.02 (-0.28, 0.32) | 0.892 |
| Religion (Muslim vs other) | 0.29 (-0.50, 1.07) | 0.477 |
| **Model statistics:** R² = 0.044; Adjusted R² = 0.037; F = 6.50; p < 0.001. | | |
